# Standardizing the measurement of maternal morbidity: Pilot study results

**DOI:** 10.1002/ijgo.12464

**Published:** 2018-05-23

**Authors:** Maria Barreix, Kelli Barbour, Affette McCaw‐Binns, Doris Chou, Max Petzold, Gathari N. Gichuhi, Luis Gadama, Frank Taulo, Özge Tunçalp, Lale Say, the WHO Maternal Morbidity Working Group (MMWG), Jose G. Cecatti, Maria L. Costa, Sara Cottler, Olubukola Fawole, Tabassum Firoz, Veronique Filippi, Atf Ghérissi, Gill Gyte, Michelle Hindin, Anoma Jayathilaka, Amanda Kalamar, Yacouba Kone, Nenad Kostanjsek, Isabelle Lange, Laura A. Magee, Arvind Mathur, Mark Morgan, Stephen Munjanja, Elizabeth Sullivan, Rachel Vanderkruik, Peter von Dadelszen

**Affiliations:** ^1^ UNDP–UNFPA–UNICEF–WHO–World Bank Special Programme of Research Development and Research Training in Human Reproduction (HRP) Department of Reproductive Health and Research WHO Geneva Switzerland; ^2^ Department of Obstetrics and Gynecology University of Utah Salt Lake City UT USA; ^3^ Department of Community Health and Psychiatry University of the West Indies Mona, Kingston Jamaica; ^4^ Center for Applied Biostatistics University of Gothenburg Gothenburg Sweden; ^5^ Maternal and Child Survival Program Jhpiego – Kenya Nairobi Kenya; ^6^ Department of Obstetrics and Gynecology College of Medicine University of Malawi Blantyre Malawi

**Keywords:** Antenatal care, Interpersonal violence, Maternal morbidity, Measurement, Nonsevere maternal morbidity, Postpartum care, Pregnancy and puerperium

## Abstract

**Objective:**

To field test a standardized instrument to measure nonsevere morbidity among antenatal and postpartum women.

**Methods:**

A cross‐sectional study was conducted in Jamaica, Kenya, and Malawi (2015–2016). Women presenting for antenatal care (ANC) or postpartum care (PPC) were recruited if they were at least 28 weeks into pregnancy or 6 weeks after delivery. They were interviewed and examined by a doctor, midwife, or nurse. Data were collected and securely stored electronically on a WHO server. Diagnosed conditions were coded and summarized using ICD‐MM.

**Results:**

A total of 1490 women (750 ANC; 740 PPC) averaging 26 years of age participated. Most women (61.6% ANC, 79.1% PPC) were healthy (no diagnosed medical or obstetric conditions). Among ANC women with clinical diagnoses, 18.3% had direct (obstetric) conditions and 18.0% indirect (medical) problems. Prevalences among PPC women were lower (12.7% and 8.6%, respectively). When screening for factors in the expanded morbidity definition, 12.8% (ANC) and 11.0% (PPC) self‐reported exposure to violence.

**Conclusion:**

Nonsevere conditions are distinct from the leading causes of maternal death and may vary across pregnancy and the puerperium. This effort to identify and measure nonsevere morbidity promotes a comprehensive understanding of morbidity, incorporating maternal self‐reporting of exposure to violence, and mental health. Further validation is needed.

## Abstract 2

A pilot study of a novel instrument to holistically measure maternal morbidity found that nonsevere conditions are distinct from the leading causes of maternal death.

## 1 INTRODUCTION

As global attention shifts from surviving pregnancy and childbirth to ensuring that women thrive throughout their lives, much remains to be done to ensure that women have a positive pregnancy experience, and to lessen the risks of pregnancy and childbirth that can lead to harmful consequences. Within the construct of Sustainable Development Goal (SDG) target 3.1 within SDG 3, and the Global Strategy to End Preventable Maternal Mortality (EPMM),^1^, ^2^ improving the measurement of maternal health will be key. Building on the success of the WHO in defining and measuring maternal near‐miss events,^3^ which also established parameters for quality of care for severe maternal complications/morbidities, action is now focused on standardizing and measuring non‐life‐threatening maternal morbidity.

As maternal mortality trends downward, measuring morbidity will be critical to monitoring the quality of maternal health care. Based on previous efforts of the WHO Department of Reproductive Health and Research (RHR) to standardize the measurement and reporting of maternal mortality and severe maternal morbidity, efforts are expanding to also address measurement of non‐life‐threatening (nonsevere) maternal morbidity, especially given that the often‐cited maternal morbidity estimate of around 20–30 morbidities for every maternal death is “not based on standard, well documented, and transparent methodologies”.^4^

RHR implemented a 5‐year project to address the lack of a scientific basis for defining, estimating, and monitoring the magnitude of maternal morbidity. It was envisioned that in addition to standardizing what is called maternal morbidity and how it is measured, doing so would not only assist program managers and policy makers to better monitor maternal morbidity, but it would also bring attention and resources to enhancing care for pregnant and postpartum women. The present paper describes the initial performance of this new set of standardized maternal morbidity measurement tools, and represents the final step in a larger initiative (described in the methods section) that seeks to advocate for and improve women's health.

The pilot study sought to field test a comprehensive instrument to measure nonsevere morbidity among women in antenatal care (ANC) and postpartum care (PPC). The paper presents study findings and insights into its future use. Subobjectives included:

1. Describing the sociodemographic characteristics of the women recruited, by country.
2. Examining the contributory factors and clinical indicators identified and their relationship to obstetric and medical diagnoses among pregnant and postpartum women.
3. Exploring the feasibility and challenges associated with administering the instrument (for research purposes only).

## 2 MATERIALS AND METHODS

This study is the culmination of a 5‐year initiative led by the Maternal Morbidity Working Group (MMWG) convened by RHR, the details of which have been published elsewhere.^4^, ^5^, ^6^ In brief, the group, composed of technical experts in maternal and women's health, began by defining non‐life‐threatening (hereafter referred to as nonsevere) maternal morbidities as: “Any health condition attributed to and/or complicating pregnancy and childbirth that has a negative impact on the woman's wellbeing and/or functioning”.^4^

To operationalize this definition, a maternal morbidity matrix was developed.^4^ It outlined three dimensions: (1) WHO's conceptual framework for the application of the International Statistical Classification of Diseases and Related Health Problems 10th Revision (ICD‐10) to deaths during pregnancy, childbirth, and the puerperium (ICD‐Maternal Mortality [ICD‐MM])^7^; (2) measurement of functional impact and disability^8^; and (3) evaluation of maternal physical and mental health, and social history.

For this pilot, the aforementioned matrix was translated into two comprehensive questionnaires: the ANC and PPC maternal morbidity measurement instruments. The tools sought a holistic view of maternal health, guided by women's perspectives. Previously validated scales were incorporated, where available, to measure mental health (General Anxiety Disorder, 7 item (GAD‐7),^9^ and the Personal Health Questionnaire, 9 item (PHQ‐9)^10^); health‐related functioning, or the ability to carry out daily tasks and social responsibilities (using the WHO Disability Assessment Schedule (WHODAS 2.0) 12‐item version^11^); sexual satisfaction^12^; substance use/abuse^13^; and exposure to violence.^14^

Both instruments were hour‐long questionnaires consisting of a patient interview, physical examination, and record review.^15^ The interview documented socioeconomic status, medical and obstetric history, and clinical symptoms. The physical examination, conducted by a healthcare professional, evaluated clinical signs. The record review extracted information on selected laboratory tests and results.

To field test the instruments, a cross‐sectional study was conducted in three countries (Jamaica, Kenya, and Malawi) in late 2015 to early 2016, over 3‐month periods in each country. All sites (n=13) were public facilities: nine in Jamaica (6 health centers and 3 referral hospitals), three in Kenya (2 district and 1 referral hospital), and one in Malawi (referral hospital). Efforts were made to include a range of facilities (primary, secondary, and tertiary referral facilities; in urban and rural settings) from different subnational areas, although the choice also depended on availability of the relevant resources to ensure high‐quality data collection. Ethical approval was obtained from the WHO Ethical Review Committee and relevant entities in each country. To describe the different types of morbidity, and enable stratification by country setting and time of administration, a sample size of 500 women per country (250 each, ANC and PPC) was calculated to be adequate. Without pooling data across sites or populations, we estimated a 6% margin of error.

Participants were conveniently selected from among women presenting for routine ANC or PPC services. Inclusion criteria were that ANC women were at least 28 weeks pregnant, and PPC women were 6–12 weeks after delivery.^15^ Women whose pregnancies ended in abortion or miscarriage were excluded, while those who experienced stillbirths were included. Women provided written informed consent before being interviewed.

Health professionals (nurses, midwives, or doctors, depending on the site) participated in a 2‐day training session to administer the ANC and PPC instruments. They were informed about the study objectives, introduced to the instruments, learned how to use the digital tablets, and gained practical experience before final selection. A manual detailing standard operating procedures for each question was developed and modified for each country. In Jamaica and Kenya, the interviewers were facility staff recruited for this project. In Malawi, ANC nurse midwives conducted the physical exam, while recent medical graduates were hired specifically to conduct the interview portion of the tool.

The instruments were developed in digital format and translated into local languages at each site. Data were gathered on tablets using OpenDataKit (University of Washington, USA) open‐source software. The digital tool limited the prevalence of missing values as interviewers could not advance until an answer was inputted. For women presenting with a condition that warranted referral (mental health score, report of violence, need for further testing, or for procedures for a certain condition), interviewers were provided with referral information on their tablets (such as the department to contact). Data were stored and managed on a secure, password‐protected, cloud‐based server system owned by WHO. Research coordinators reviewed and approved the submitted electronic records for completeness and quality.

Conditions diagnosed by the health team were recorded as open‐ended questions, then coded by a clinical officer (KB), and summarized using ICD‐10 and ICD‐MM guidelines. Obstetric (direct) complications were grouped, using ICD‐MM categories, into: hypertensive disorders, obstetric hemorrhage, pregnancy‐related infection, and other specific direct conditions. Indirect (medical) conditions were classified by organ system, namely cardiovascular, hematologic, endocrine, and other medical conditions. Injuries (unintentional or otherwise) were classified separately. Contributory factors considered included obesity (body mass index ≥30), and self‐reported experiences such as exposure to violence, anxiety (GAD‐7 score ≥10), and depression (PHQ‐9 score ≥10). To document prevalence of medical conditions, the healthcare provider's diagnoses at the time of interview were the gold standard; however, mental health diagnoses were based on results in the standardized GAD (anxiety) and PHQ (depression) questionnaires.

To demonstrate the utility of the tool for research and future health‐service intervention, multivariate (logistic regression) analyses were undertaken. Covariate variables were examined by the following groupings.

1. Contributory factors: substance use, not having a partner, being obese, and being sexually dissatisfied.
2. Equity/access: receiving care at a primary or referral facility, urban residence, and having only primary level education.
3. Demographic characteristics: age (≥35 years, or ≤20 years), and parity (primiparous vs multiparous).

Data were compared among countries using the χ^2^ test, Fisher exact test, or Kruskal‐Wallis test, as appropriate. Forward and backward regressions were employed based on *P*≤0.10. Statistical significance was considered to be *P*≤0.5. Confidence intervals for continuous data were based on normal distribution approximation. Statistical analyses were conducted in Stata/SE version 14.1 (StataCorp LLC, College Station, TX, USA).

## 3 RESULTS

Interviews were conducted with 1490 women (750 ANC; 740 PPC). Sixteen ANC and 39 PPC women declined to participate. While similar to the study population, ANC refusers were older (27.6 ± 8.4 vs 26.2 ± 5.9 years, *P*<0.001) and PPC refusers were more likely to have a partner (86.8% vs 69.3%, *P*<0.001).

Both the ANC and PPC study populations averaged 26 years of age (Tables 1 and 2). Fewer African women were having their first child (range, 27.7%–38.8% in both groups) compared with Jamaicans (almost 44% in both groups). Only six (0.8%) PPC women interviewed reported stillbirths. In Kenya (79.5% ANC; 82.2% PPC) and Malawi (92.5% ANC; 89.3% PPC), most women reported having a partner, while in Jamaica only 41.9% of ANC and 38.3% of PPC women reported having a partner. Jamaican women were also least likely to be illiterate (0.8% ANC; 2.0% PPC). While most ANC women were employed (52.4%), this declined to 48.1% among PPC women. Most participants (85.1% ANC, *P*<0.001, and 72.8% PPC, *P*<0.001) were recruited from referral sites to ensure that the instrument could be tested on women with a morbid condition in pregnancy and the puerperium.

**Table 1:** Characteristics of the antenatal care study population.^a^^,^ b

Antenatal care (ANC)	Total (n=750)	Jamaica (n=253)	Kenya (n=258)	Malawi (n=239)	*P* value
Maternal age, y	26.2 ± 5.9	25.8 ± 6.2	25.1 ± 5.5	27.7 ± 5.6	0.02
<20	91 (12.1)	37 (14.6)	36 (14.0)	18 (7.5)	
20–34	591 (78.8)	193 (76.3)	206 (79.4)	192 (80.3)	
≥35	68 (9.1)	23 (9.1)	16 (6.20)	29 (12.1)	
Marital status					<0.001
No partner	218 (29.1)	147 (58.1)	53 (20.5)	18 (7.5)	
Has partner	532 (70.9)	106 (41.9)	205 (79.5)	221 (92.5)	
Education					<0.001
Primary or less	202 (26.9)	15 (5.9)	125 (48.5)	62 (25.9)	
Secondary	370 (49.3)	172 (68.0)	100 (38.8)	98 (41.0)	
Higher	178 (23.7)	66 (26.1)	33 (12.8)	79 (33.1)	
Literacy^c^					<0.001
Cannot read	28 (3.8)	2 (0.8)	10 (3.9)	16 (6.7)	
Can read parts of sentence	44 (5.9)	11 (4.4)	31 (12.4)	2 (0.8)	
Can read whole sentence	670 (90.3)	240 (94.9)	210 (83.7)	220 (92.4)	
Employed					0.158
No	357 (47.6)	114 (45.1)	117 (45.4)	126 (52.7)	
Yes	393 (52.4)	139 (54.9)	141 (54.7)	113 (47.3)	
Travel time to facility, min					<0.001
<15	100 (13.3)	69 (27.3)	17 (7.0)	14 (5.9)	
15–30	321 (42.8)	106 (41.9)	144 (55.8)	71 (29.7)	
30–60	198 (26.4)	54 (21.3)	70 (27.1)	74 (30.1)	
>60	131 (17.5)	24 (9.5)	27 (10.5)	80 (33.5)	
Interview site					<0.001
Community clinics	112 (14.9)	112 (44.3)	0 (0.0)	0 (0.0)	
ANC clinic at hospital	638 (85.1)	141 (55.7)	258 (100.0)	239 (100.0)	
Parity	1.3 ± 1.6	1.2 ± 1.6	1.5 ± 1.4	1.3 ± 1.5	0.002
0	271 (36.1)	111 (43.9)	77 (29.8)	83 (34.7)	
1	207 (27.6)	68 (26.9)	69 (26.7)	70 (29.3)	
2–4	237 (31.6)	58 (22.9)	101 (39.2)	78 (32.6)	
≥5	35 (4.7)	16 (6.3)	11 (4.3)	8 (3.4)	

a All *P* values refer to χ^2^ results, unless cell values were <5 and Fisher exact or Kruskal‐Wallis tests were employed depending on the variable.

b Values are given as mean ± SD or number (percentage) unless otherwise stated.

c Excludes 1 blind woman and 7 for whom the correct language card was missing.

**Table 2:** Characteristics of the postpartum care study population.^a^^,^ b

Postpartum care (PPC)	Total (n=740)	Jamaica (n=256)	Kenya (n=242)	Malawi (n=242)	*P* value
Maternal age, y	25.8 ± 6.3	26.1 ± 6.5	24.7 ± 5.7	26.5 ± 6.3	0.272
<20	115 (15.5)	38 (14.8)	43 (17.8)	34 (14.1)	
20–34	550 (74.3)	186 (72.7)	182 (75.2)	182 (75.2)	
≥35	75 (10.1)	32 (12.5)	17 (7.0)	26 (10.7)	
Marital status					<0.001
No partner	227 (30.7)	158 (61.7)	43 (17.8)	26 (10.7)	
Has partner	513 (69.3)	98 (38.3)	199 (82.2)	216 (89.3)	
Education					<0.001
Primary or less	214 (28.9)	27 (10.6)	117 (48.4)	70 (28.9)	
Secondary	356 (48.8)	135 (52.7)	99 (40.9)	122 (50.4)	
Higher	170 (22.3)	94 (36.7)	26 (10.7)	50 (20.7)	
Literacy.^c^					<0.001
Cannot read	32 (4.3)	5 (2.0)	7 (2.9)	20 (8.3)	
Can read parts of sentence	47 (6.4)	10 (3.9)	27 (11.3)	11 (4.1)	
Can read whole sentence	658 (89.3)	241 (94.1)	205 (85.8)	212 (87.6)	
Employed					0.005
No	328 (51.9)	119 (46.5)	119 (49.2)	146 (60.3)	
Yes	356 (48.1)	137 (53.5)	123 (50.8)	96 (36.7)	
Travel time to facility, min					<0.001
<15	163 (22.0)	119 (46.5)	22 (9.1)	22 (9.1)	
15–30	279 (37.7)	92 (35.9)	121 (50.0)	66 (27.3)	
30–60	191 (25.8)	28 (10.9)	75 (31.0)	88 (36.4)	
>60	107 (14.5)	17 (6.6)	24 (9.9)	66 (27.3)	
Interview site					<0.001
Community clinics	201 (27.2)	201 (78.5)	0 (0.0)	0 (0.0)	
PPC clinic at hospital	539 (72.8)	55 (21.5)	242 (100.0)	242 (100.0)	
Parity	2.3 ± 1.5	2.2 ± 1.5	2.5 ± 1.5	2.3 ± 1.4	0.005
1	273 (36.8)	112 (43.8)	67 (27.7)	94 (38.8)	
2–4	405 (54.6)	126 (49.2)	153 (63.2)	126 (52.1)	
≥5	62 (8.4)	18 (7.0)	22 (9.1)	22 (9.1)	

a All *P* values refer to χ^2^ results, unless cell values were <5 and Fisher exact or Kruskal‐Wallis tests were employed depending on the variable.

b Values are given as mean ± SD or number (percentage) unless otherwise stated.

c Excludes 3 women who were blind.

In expanding the definition of morbidity, selected contributory factors were explored. We examined exposure to violence by asking women whether they had been “afraid of your current/most recent husband or partner or anyone else,” or whether “since pregnancy/delivery, was there ever a time when you were pushed, slapped, hit, kicked or beaten by (any of) your husband/partner(s) or anyone else?” If women replied affirmatively to either question, they were asked three additional violence‐related questions. Thirteen percent (12.8%; n=96, *P*=0.018) of ANC and 11.0% (n=81, *P*<0.001) of PPC women reported being afraid of, or having experienced some form of physical violence from, their current partner or someone else, with rates varying by site from 7% to 17%. We also explored substance use through self‐reporting (3%–4% overall; Table 3) and, given the global prevalence of obesity, women's heights and weights were documented (Table 4).

**Table 3:** Women's social risk factors in the antenatal care and postpartum care study populations.^a^^,^ b

	Total	Jamaica	Kenya	Malawi	*P* value
Antenatal care	(n=750)	(n=253)	(n=258)	(n=239)	
Substance use^c^					0.004
No	720 (96.0)	236 (93.3)	253 (98.1)	231 (96.7)	
Yes	30 (4.0)	17 (6.7)	5 (1.9)	8 (3.4)	
Exposure to violence^d^					0.018
No	652 (86.9)	222 (87.8)	211 (82.4)	219 (91.6)	
Yes	96 (12.8)	31 (12.3)	45 (17.4)	20 (8.4)	
Postpartum care	(n=740)	(n=256)	(n=242)	(n=242)	
Substance use^c^					<0.001
No	717 (96.9)	236 (92.2)	242 (100.0)	239 (98.8)	
Yes	22 (3.0)	20 (7.8)	0 (0.0)	2 (0.8)	
Exposure to violence^d^					<0.001
No	655 (88.5)	232 (90.6)	225 (93.0)	198 (82.9)	
Yes	81 (11.0)	24 (9.4)	40 (16.5)	17 (7.0)	

a All *P* values refer to χ^2^ results, unless cell values were <5 and Fisher exact or Kruskal‐Wallis tests were employed depending on the variable.

b Values are given as number (percentage) unless otherwise indicated.

c Defined as use of the following substances: tobacco products, alcoholic beverages, marijuana (ganja), inhalants.

d Women who responded no or never to the following questions: (1) Are you afraid of your current/most recent husband or partner or anyone else? Would you say never, sometimes, many times, most/all of the time?; (2) Since pregnancy/delivery, was there ever a time when you were pushed, slapped, hit, kicked, or beaten by (any of) your husband/partner(s) or anyone else?

**Table 4:** Prevalence of obesity, number, and leading conditions identified during antenatal and postpartum care visits, by country.^a^^,^ b

	Total	Jamaica	Kenya	Malawi	*P* value
Antenatal care	(n=750)	(n=253)	(n=258)	(n=239)	
Obesity					
Body mass Index ≥30	258 (34.9)	114 (45.4)	53 (21.0)	91 (38.4)	<0.001
Number of conditions diagnosed					0.018
0	462 (61.6)	139 (54.9)	167 (64.7)	156 (65.3)	
1	205 (27.3)	69 (27.3)	73 (28.3)	63 (26.4)	
2	65 (8.7)	38 (15.0)	14 (5.4)	13 (5.4)	
3–6	18 (2.4)	7 (2.7)	4 (1.6)	7 (2.9)	
Categories of conditions					
Direct	137 (18.3)	31 (12.3)	42 (16.3)	64 (26.8)	<0.001
Indirect	135 (18.0)	62 (24.5)	49 (19.0)	24 (10.4)	<0.001
Exposure to violence	96 (12.8)	31 (12.3)	45 (17.4)	20 (8.4)	0.004
Leading direct conditions					
Pregnancy infections	78 (10.4)	6 (2.4)	27 (10.5)	45 (18.8)	<0.001
Hypertensive disorders	22 (2.9)	9 (3.6)	1 (0.4)	12 (5.0)	0.002
Fetus growth/length anomaly	12 (1.6)	6 (2.4)	3 (1.2)	3 (1.3)	0.581
Other	7 (0.9)	4 (1.6)	0 (–)	3 (1.3)	0.105
Obstetric trauma	5 (0.7)	3 (1.2)	1 (0.4)	1 (0.4)	0.541
Leading indirect conditions					
Sexually transmitted infection, vaginitis	51 (6.8)	20 (7.9)	17 (6.6)	14 (5.9)	0.657
Psychiatric	48 (6.4)	40 (15.8)	6 (2.3)	2 (0.8)	<0.001
Hematologic	28 (3.7)	10 (4.0)	12 (4.7)	6 (2.5)	0.442
Malaria	9 (1.2)	0 (–)	9 (3.5)	0 (–)	<0.001
Respiratory	6 (0.8)	0 (–)	5 (1.9)	1 (0.4)	0.043
Postpartum care	(n=740)	(n=256)	(n=242)	(n=242)	
Obesity					
Body Mass Index ≥30	164 (22.6)	94 (37.3)	32 (13.5)	38 (16.1)	<0.001
Number of conditions diagnosed					0.027
0	585 (79.1)	184 (71.9)	195 (80.6)	206 (85.1)	
1	112 (15.1)	51 (19.9)	31 (12.8)	30 (12.4)	
2	32 (4.3)	15 (5.7)	11 (4.6)	6 (2.5)	
3–4	11 (1.4)	6 (2.4)	5 (1.1)	0 (–)	
Categories of conditions					
Direct	64 (8.7)	19 (7.4)	15 (6.2)	30 (12.4)	0.036
Indirect	94 (12.7)	51 (19.9)	36 (14.9)	7 (2.9)	<0.001
Exposure to violence	81 (11.0)	24 (9.4)	40 (16.5)	17 (7.0)	<0.001
Leading direct conditions					
Hypertensive disorders	30 (4.1)	12 (4.7)	4 (1.7)	14 (5.8)	0.43
Pregnancy infections	28 (3.8)	4 (1.6)	9 (3.7)	15 (6.2)	0.024
Obstetric hemorrhage	2 (0.3)	1 (0.4)	0 (–)	1 (0.4)	0.999
Wound complication	2 (0.3)	1 (0.4)	0 (–)	1 (0.4)	0.999
Other	1 (0.1)	1 (0.4)	0 (–)	0 (–)	0.999
Leading indirect conditions					
Sexually transmitted infection, vaginitis	48 (6.5)	38 (14.8)	6 (2.5)	4 (1.7)	<0.001
Psychiatric	16 (2.2)	13 (5.1)	2 (0.8)	1 (0.4)	<0.001
Hematological	13 (1.8)	3 (1.2)	9 (3.7)	1 (0.4)	0.024
Gastrointestinal	5 (0.7)	1 (0.4)	4 (1.7)	0 (–)	0.088
Other infection	4 (0.5)	2 (0.8)	2 (0.8)	0 (–)	0.554

a All *P* values refer to χ^2^ results, unless cell values were <5 and Fisher exact or Kruskal‐Wallis tests were employed depending on the variable.

b Values are given as number (percentage) unless otherwise indicated.

Most ANC (61.6%, *P*=0.018) and PPC (79.1%, *P*=0.027) women were healthy (i.e. they were not diagnosed with any conditions by healthcare providers, and did not self‐report mental health problems). Twenty‐seven percent (27.3%, n=205) of ANC and 15.1% (n=112) of the PPC population were diagnosed with one morbidity (including screening positive for depression or anxiety), and 11.1% (n=83) of ANC and 5.7% (n=43) of PPC women had two or more conditions (Table 5). ANC women were almost equally diagnosed with direct (18.3%, n=137, *P*<0.001) or indirect conditions (18.0%, n=135, *P*<0.001), whereas PPC women were more likely to be diagnosed with indirect (12.7%, *P*<0.001) than with direct conditions (8.7%, *P*=0.036).

**Table 5:** Health status reported by women attending antenatal and postpartum care visits.^a^^,^ b

	Total	Jamaica	Kenya	Malawi	*P* value
Antenatal care	(n=750)	(n=253)	(n=258)	(n=239)	
Overall health rating					0.005
Very good	165 (22.0)	72 (28.5)	23 (8.9)	70 (29.3)	
Good	411 (54.8)	131 (51.8)	177 (68.6)	103 (43.1)	
Neither poor nor good	136 (18.1)	37 (14.6)	52 (20.2)	47 (19.7)	
Poor	36 (4.8)	12 (4.7)	6 (2.3)	18 (7.5)	
Very poor	2 (0.3)	1 (0.4)	0 (0.0)	1 (0.4)	
Have you been told you have anything wrong/any medical condition(s)?					0.034
No	641 (85.5)	213 (84.2)	232 (89.9)	196 (82.1)	
Yes	109 (14.5)	40 (15.8)	26 (10.1)	43 (18.0)	
Are you taking any medication(s) today?					0.036
No	181 (24.1)	67 (26.5)	48 (18.6)	66 (27.6)	
Yes	569 (75.9)	186 (73.5)	210 (81.4)	173 (72.4)	
Do you have any other medical conditions or other problem(s) you would like to report?					0.611
No	650 (86.7)	215 (85.0)	225 (87.2)	210 (87.8)	
Yes	100 (13.3)	38 (15.0)	33 (12.8)	29 (12.1)	
Anxiety score	2.6 ± 3.1	3.6 ± 3.8	2.1 ± 2.5	1.6 ± 2.0	<0.001
Depression score	2.4 ± 3.3	4.0 ± 4.3	2.0 ± 2.3	1.1 ± 1.9	<0.001
Postpartum care	(n=740)	(n=256)	(n=242)	(n=242)	
Overall health rating^c^					0.001
Very good	240 (32.4)	102 (39.8)	34 (14.1)	104 (43.0)	
Good	418 (56.5)	131 (51.2)	177 (73.1)	110 (45.5)	
Neither poor nor good	66 (8.9)	17 (6.6)	27 (11.2)	22 (9.1)	
Poor	16 (2.2)	6 (2.3)	4 (1.7)	6 (2.5)	
Have you been told you have anything wrong/any medical condition(s)?					0.098
No	695 (93.9)	234 (91.4)	229 (94.6)	232 (95.9)	
Yes	45 (6.1)	22 (8.6)	13 (5.4)	10 (4.1)	
Are you taking any medication(s) today?					<0.001
No	520 (70.3)	150 (58.6)	159 (65.7)	211 (87.2)	
Yes	220 (29.7)	106 (41.4)	83 (34.3)	31 (12.8)	
Do you have any other medical conditions or other problem(s) you would like to report?					0.012
No	673 (91.0)	227 (88.7)	215 (88.8)	231 (95.5)	
Yes	67 (9.1)	29 (11.3)	27 (11.2)	11 (4.6)	
Anxiety score	1.52 ± 2.4	2.3 ± 2.9	1.5 ± 2.3	0.6 ± 1.5	<0.001
Depression score	1.2 ± 2.0	2.1 ± 2.7	1.0 ± 1.6	0.3 ± 0.9	<0.0001

a All *P* values refer to χ^2^ results, unless cell values were <5 and Fisher exact or Kruskal‐Wallis tests were employed depending on the variable.

b Values are given as number (percentage) or mean ± SD unless otherwise indicated.

c No PPC women reported very poor overall health.

Using ICD‐MM direct condition groups, ANC women commonly presented with pregnancy‐related infections (10.4%, n=78, *P*<0.001), hypertensive disorders (2.9%, n=22, *P*=0.002), and fetal growth/length anomalies (1.6%, n=12, *P*=0.581). The hypertensive disorders category combines pre‐eclampsia, gestational hypertension, and clinical observations of “elevated blood pressure”. The most prevalent PPC direct conditions were hypertensive disorders (4.1%, n=30, *P*=0.43) and pregnancy‐related infections (3.8%, n=28, *P*=0.024). For both ANC and PPC women, the most common indirect conditions were sexually transmitted infections, vaginitis (6.8%, n=51, *P*=0.657; 6.5%, n=48, *P*<0.001), and psychiatric conditions (6.4%, n=48, *P*<0.001; 2.2%, n=16, *P*<0.001). Clinical examination documented four women with depression in the ANC population, and two women in the PPC population (no women were diagnosed with anxiety by a healthcare provider). Conversely, the GAD and PHQ found that 6.3% (n=47) of ANC and 2.2% (n=16) of PPC women had one or both signs of significant distress (Table 5). The psychiatric conditions category therefore incorporated diagnosis by a healthcare provider, or scoring 10 or above on the GAD‐7 or PHQ‐9 questionnaires.

We conducted univariate and logistical analyses for ANC and PPC women to evaluate the associations of contributory and demographic factors, if any, with diagnosis of any morbidity (including self‐reported mental health conditions). The univariate analysis showed that for ANC women, substance use (*P*=0.023), obesity (*P*=0.008), being sexually dissatisfied (*P*=0.001) or being under 20 years of age (*P*=0.056) were associated with a morbidity diagnosis of any kind. For PPC women, not having a partner (*P*=0.054), rural residence (*P*=0.043), and being sexually dissatisfied (*P*<0.001) were associated with a morbidity diagnosis.

When all factors were included in a backward regression, only sexual dissatisfaction (ANC: OR 1.53, 95% CI, 1.1–2.1, *P*=0.015; PPC: OR 1.88, 95% CI, 1.2–2.9, *P*=0.004), and living in an urban setting were independently associated with any morbidity diagnosis, with urban residence being somewhat protective (PPC only: OR 0.75, 95% CI, 0.5–1.1, *P*=0.097).

While time and resources did not permit a rigorous qualitative evaluation of the feasibility and challenges associated with administering the tools for research purposes, we did discuss the process with data collectors at feedback meetings. The relatively low refusal rate was an indication that women were willing to participate in the process. During discussions with data collectors to determine the feasibility and challenges of implementing the tool, it became clear that while women were open to talking about difficult topics (violence, mental health, and sexual satisfaction) that were not generally associated with routine care, interviewers (trained health workers) felt unprepared to engage in these discussions, or were concerned that there were inadequate referral services for women presenting with these issues/conditions. Interviewers did agree, however, that these were important issues and need to be considered in future clinical interactions with women, especially the issue of depression.

## 4 DISCUSSION

The MMWG's efforts have led to the development of a measurement instrument to describe nonsevere maternal morbidity in a manner that highlights the woman's experience of pregnancy as the starting point. Its woman‐centered questions have allowed for self‐reporting^15^ of factors contributing to morbidity. The instrument is then strengthened by the clinical perspective through diagnoses by skilled providers. The pilot study aimed to field test this comprehensive instrument, which aims to standardize the measurement of nonsevere morbidity among antenatal and postpartum women. The piloted instrument measured morbidity more broadly by incorporating a wide range of self‐reported factors, beginning with symptoms, contributing factors, and ability to carry out daily activities (functioning). Additionally, based on study results, we described the sociodemographic characteristics of participating women, as well as estimating both contributory factors and the prevalence of maternal morbidities/conditions, and their relationship. These represent areas for further exploration and strategic development.

Of the approximately 210 million pregnancies that occur every year, around 303 000 culminate in a maternal death, yet there is no consensus on how many women suffer non‐life‐threatening complications,^16^, ^17^ probably due to the wide variety of previous definitions.^18^, ^19^ Additionally, Koblinsky et al.^20^ have described the reliability issues associated with self‐reporting versus diagnoses by community or skilled care providers (“gold standard”). Our findings depict a similar phenomenon, where women's reported exposures yielded higher prevalence rates than those based on clinical assessment alone, such as when mental health and exposure to violence were evaluated.

We believe that addressing symptoms and conditions that women identify as rendering them less able to function, or causing significant discomfort, will be fundamental to addressing nonsevere maternal morbidity. The challenge, in a setting with limited resources, is to improve the health and social service system's capacity to respond to the demand for care that identifying and addressing these issues may require. As such, the purpose of the instrument needs to be clear for users, and its design should allow persons to utilize those components that address their needs. The instrument may need to be designed in modules that measure separately those clinical problems that health systems should be prepared to assist patients to manage, and issues for investigation by specifically interested researchers. However, this approach might sideline issues women deem important by relegating certain issues to lower priority.

The pilot's findings and the MMWG's broader scope of self‐reported conditions have led to a rethinking of the concept of maternal morbidity as a whole. Traditionally, the progression from healthy pregnancy to severe maternal morbidity and mortality has been presented as a linear or iceberg concept, which does not fully capture or reflect certain non‐life‐threatening conditions that could complicate a woman's life long term. The pilot results support a more cyclical (nonlinear) perspective on maternal morbidity, an idea that is further expanded in the paper by Filippi et al.^21^ in this Supplement.

It is interesting to note that medical problems had similar levels of prevalence as obstetric ones in ANC women, and accounted for the majority of PPC diagnoses in Jamaica and Kenya. This highlights changes in demographic and health status, with the surge of pre‐existing noncommunicable diseases in parallel with the high prevalence of obesity and related medical comorbidities. It may also indicate that while the general emphasis of ANC has been on obstetric conditions with high case‐fatality rates, such as obstetric hemorrhage, this approach may fail to address the universal health needs of women. Many women experience their first contact with health services when they become pregnant, and this opportunity should be exploited to improve their overall health.

Validation exercises are needed to determine the efficacy of the instrument in accurately identifying the burden of disease in other settings and populations. Defining hypertension in pregnancy broadly, a Ghanaian study documented an 11.3% prevalence of the hypertensive disorders of pregnancy^22^—a much higher rate than this study. It is not clear what the possible influence is in case ascertainment of resource constraints such as access to equipment, supplies, and diagnostic skills.

Research has shown that the prevalence of physical violence varies significantly by setting. Rates are generally higher (15%–71%), however, than those found in our study.^14^ This may be due to the tool having only two screening questions, and to their broad scope. When women are asked specific questions about their experience with violence, rather than more general questions (i.e. “are you afraid…?”), they are more likely to respond affirmatively. Yet it is relevant to address exposure to violence, especially intimate partner violence, as it has been linked to poor ANC utilization,^23^ low birth weight, and preterm delivery,^24^ and possibly pregnancy‐associated suicide and homicide.^25^

Similarly, our clinical examination, and self‐reporting of depression and anxiety (using GAD‐7 and PHQ‐9 for screening), documented a low prevalence. In many settings, mental health concerns are highly stigmatized. Cases may be missed as patients may somaticize these concerns through vague symptoms such as fatigue.^26^ A Ghanaian study utilizing the PHQ in a postnatal population found a rate (3.5%; 95% CI, 3.2–3.7) that mirrored the one in our Jamaican population (3.5%; 95% CI, 1.3–5.8).^27^ Many social and economic factors have been linked to antenatal and postnatal depression, including experiencing a first pregnancy,^28^ being unmarried,^29^ exposure to intimate partner violence,^30^ and lack of partner/baby father support,^31^ or of family cohesion.^28^ Identifying and treating these mothers is critical not only to their health but also to the survival and development of their infants.^32^ The documented rates require refocusing ANC and PPC services to ensure that these women receive appropriate care.

Limitations of the pilot study included a nonrepresentative sample population, especially in Kenya and Malawi, which was drawn from large urban hospitals. Given this relatively healthy population of women, we had to aggregate many diagnoses, as numbers were too small to separate specific diagnoses. The instrument does not include questions addressing the baby's signs and symptoms, or complications of labor and delivery, which may also help identify morbidity in mothers. Additionally, we were unable to follow the same women over time to measure the temporality of conditions, or distinguish between conditions that developed during pregnancy and prior to pregnancy (i.e. women diagnosed with diabetes mellitus were aggregated into the gestational diabetes mellitus category, as most are first tested and diagnosed during pregnancy). While the current instrument's focus on women's health‐related functioning is important, it lacks a well‐being component to illuminate how women interpret the experience of pregnancy, and how they feel about their health.

The pilot study presents a novel approach to measuring nonsevere maternal morbidity, and furthers the work of WHO and the MMWG to standardize the definition, identification, and measurement process. This holistic approach to assessing maternal morbidity will provide a basis for advocacy for women's health and rights in the broader context.^33^

Further research is needed to validate the instrument, and to ensure that the data collected can be used to assess and improve maternal care, especially postpartum visits and continuing health care for women. This is consistent with the EPMM strategy to “address all causes of maternal mortality, reproductive and maternal morbidities and related disabilities”^2^ and aligns with the progress needed to achieve the targets envisioned by the Global Strategy for Women's, Children's and Adolescents’ Health 2016–2030^34^ and the SDG agenda.

## AUTHOR CONTRIBUTIONS

MB, KB, AMB, and DC led the manuscript writing process. MP conducted the data analysis. AMB, GN, FT, and LG implemented the study in each of their country settings, and edited the manuscript. OT supported the tool development and analysis process. LS conceptualized the maternal morbidity measurement initiative. All authors read and approved the final manuscript.

## MMWG MEMBERS

Jose G. Cecatti, Maria L. Costa, Sara Cottler, Olubukola Fawole, Tabassum Firoz, Veronique Filippi, Atf Ghérissi, Gill Gyte, Michelle Hindin, Anoma Jayathilaka, Amanda Kalamar, Yacouba Kone, Nenad Kostanjsek, Isabelle Lange, Laura A. Magee, Arvind Mathur, Mark Morgan, Stephen Munjanja, Elizabeth Sullivan, Rachel Vanderkruik, Peter von Dadelszen.

## CONFLICTS OF INTEREST

The authors declare no conflicts of interest.
